# The evolution of cardiac changes after breast cancer adjuvant radiotherapy – A six-year follow-up study

**DOI:** 10.1016/j.ctro.2025.101078

**Published:** 2025-11-15

**Authors:** Mikko Moisander, Suvi Tuohinen, Heidi Lähdeaho, Heini Huhtala, Kjell Nikus, Vesa Virtanen, Pirkko-Liisa Kellokumpu-Lehtinen, Pekka Raatikainen, Tanja Skyttä

**Affiliations:** a Faculty of Medicine and Health Technology, Tampere University, PO Box 100, 33014 Tampere, Finland; b Department of Oncology, Tays Cancer Center, Tampere University Hospital, PO Box 2000, 33521 Tampere, Finland; c Heart Hospital, Tampere University Hospital, PO Box 2000, 33521 Tampere, Finland; d Heart and Lung Center, Helsinki University Central Hospital and Helsinki University, PO Box 100, 00029 Helsinki, Finland; e Faculty of Social Sciences, Tampere University, PO Box 100, 33014 Tampere, Finland; f Research, Development and Innovation Center, Tampere University Hospital, PO Box 2000, 33521, Finland

**Keywords:** Cardio-oncology, Radiotherapy, Breast cancer, Cardiotoxicity, Cardiac biomarkers

## Abstract

**Introduction:**

This prospective study investigated the long-term effects of adjuvant radiotherapy (RT) on cardiac function and biomarkers in early breast cancer patients over a six-year follow-up.

**Methods:**

Seventy-three women treated with RT alone, without chemotherapy, were included. Cardiac radiation doses were quantified, and echocardiography and serum biomarkers (high-sensitivity cardiac troponin T [hscTnT] and N-terminal pro-brain natriuretic peptide [proBNP]) were assessed before RT and at three- and six-years post-treatment.

**Results:**

Cardiac biomarkers increased significantly during follow-up. Median hscTnT rose from 4 to 6 ng/L (p<0.001), correlating with higher radiation doses to the heart in left-sided patients. ProBNP increased from 78 to 118 ng/L (p<0.001). Left ventricular (LV) systolic function declined in left-sided patients: ejection fraction decreased from 65% to 60% (p=0.002), global longitudinal strain from –18% to –17% (p=0.006), and stroke volume from 74 to 67 mL (p=0.015). Diastolic dysfunction also progressed, with impairments in isovolumic relaxation time and left atrial ejection fraction. Aromatase inhibitor (AI) use and higher mean heart dose were associated with greater cardiac impairment.

**Conclusion:**

Subclinical deterioration in cardiac function and elevated biomarkers were evident three years after RT and persisted at six years, suggesting permanent cardiac effects. Both radiation dose and AI therapy contributed to these changes. These findings underscore the importance of long-term cardiac monitoring and support the use of sensitive imaging and biomarkers to detect early radiotherapy-induced cardiotoxicity.

## Highlights

• Six-year follow-up for cardiotoxicity after breast cancer adjuvant radiotherapy.
• Early subclinical myocardial changes detected by echocardiography.
• Cardiac biomarkers rose significantly.
• Aromatase inhibitor therapy and radiation dose were associated with cardiac changes.

## Graphical abstract

**Figure:**
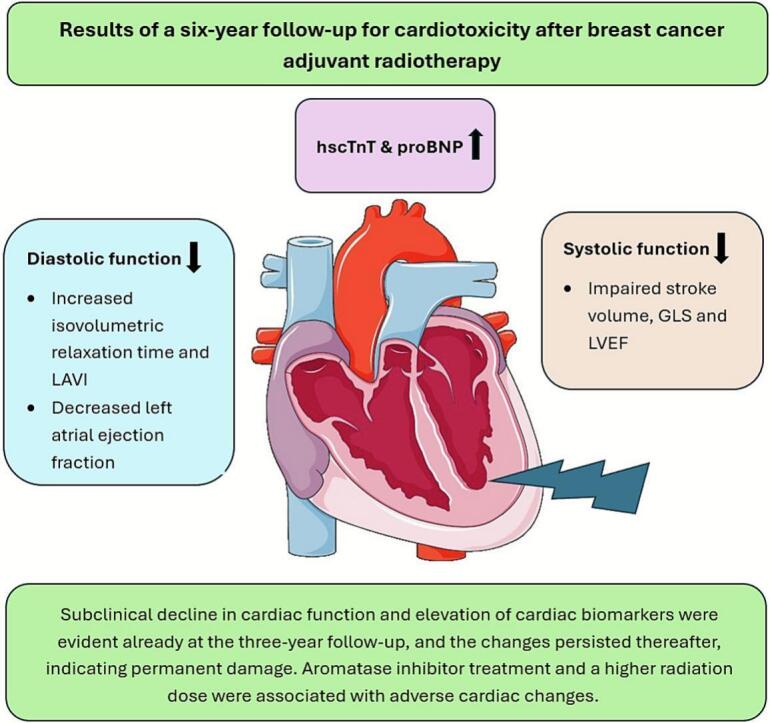

## Introduction

Breast cancer is the second most often diagnosed cancer worldwide, with an estimated 2.3 million new cases per year [1]. On the other hand, while 666,000 breast cancer-related deaths occur yearly, the prevalence of breast cancer survivors is increasing [1]. It has been estimated that globally, women have more lost disability-adjusted life years to breast cancer than any other cancer [2]. Most of early breast cancer patients will receive some adjuvant treatment (radiotherapy, chemotherapy, hormonal therapy or monoclonal antibodies), which will have diverse long-term adverse effects [3].

Radiotherapy (RT) plays a central role in the management of early-stage breast cancer, significantly improving local control and overall survival [4,5]. However, incidental radiation exposure to the heart, especially in left-sided breast cancer, has raised long-term concerns regarding cardiotoxicity [6,7]. Remarkable advances have been made in recent years in developing new techniques to reduce radiation doses to the heart and introducing them to clinical practice [8]. However, there is deficient knowledge about the evolution of cardiac changes after RT and how to predict which patients will develop a significant cardiac disease.

This study aimed to assess the six-year evolution of cardiac changes following adjuvant RT in early breast cancer patients, using serial echocardiographic and biomarker evaluations.

## Methods

### Patient population

Eighty eligible women treated with adjuvant RT after breast cancer surgery were enrolled in this prospective single-center study between June 2011 and May 2013. Adjuvant chemotherapy was an exclusion criterion to avoid additive cardiotoxic effects. Aromatase inhibitor (AI) or tamoxifen therapy was allowed when required. In addition, patients >80 years old and those with major cardiac diseases (e.g., heart failure, atrial fibrillation, or myocardial infarction) or the need for dialysis were excluded. The study complied with the Declaration of Helsinki, and the local Ethics Committee approved the study protocol (R10160 and R11149). All participants signed an informed consent form before study enrollment.

### Radiotherapy

All patients had 3D computer tomography treatment planning. RT was delivered according to the institutional clinical guidelines. The treatment doses were 50 Gy in 2 Gy fractions or 42.56 Gy in 2.66 Gy fractions. When indicated, an additional boost of 16 Gy in 2 Gy fractions to the tumor bed was given. The full details of RT planning and contouring of the cardiac structures have been described previously [9].

### Echocardiographic examinations

The full details of the echocardiographic examinations have been described previously [10]. In short, a comprehensive echocardiography study following the European guidelines [11] was performed by the same cardiologist (ST) at the baseline and at three and six years after the completion of RT.

### Cardiac biomarker analysis

High-sensitivity cardiac troponin T (hscTnT) and N-terminal pro-brain natriuretic peptide (proBNP) were analyzed at the same time points as echocardiography. While the detection limit of hscTnT was 5  ng/L, all values below this (<5 ng/l) were defined to be 4 in the analysis (lowest detection limit (LOD)/√2) [12].

### Statistical analysis

The data are presented as the means with standard deviations (SD) for variables with normal distributions, as medians with quartiles (Q_1_-Q_3_) for nonnormally distributed variables, or as numbers with percentages for categorical variables.

When appropriate, the differences between groups were tested with independent samples t-tests or Mann-Whitney U tests for continuous variables and with the chi-square or Fisher’s exact test for categorical variables. The measurement changes were tested with paired samples t-tests or Wilcoxon signed-rank tests. Correlations were estimated using Spearman’s rank correlation coefficients (ρ).

Multivariable linear regression analyses were performed to model the changes in cardiac biomarkers and echocardiographic findings, adjusting the models for breast cancer laterality, age, body mass index (BMI), hypertension, current smoking status, AI use, and mean heart dose (MHD).

The analysis was performed with IBM SPSS Statistics software, version 27.0 for Windows (Armonk, NY, USA). All p-values are two-sided; p-values less than 0.05 were considered statistically significant.

## Results

Of the 80 initially enrolled patients, 73 completed the six-year follow-up and were included in the final analysis: 55 with left-sided and 18 with right-sided breast cancer. Seven patients were excluded due to death (n = 2), withdrawal of consent (n = 2), or additional treatments following recurrence or secondary malignancies (n = 3). No patient received chemotherapy. Baseline characteristics are presented in Table 1.

**Table 1:** Baseline patient characteristics and radiation doses.

	All (n = 73)		Left-sided (n = 55)		Right-sided (n = 18)		p Value left vs. right
	Mean/median	(SD)/ Q_1_-Q_3_	Mean/median	(SD)/ Q_1_-Q_3_	Mean/median	(SD)/ Q_1_-Q_3_	
Age (years)	63	(6)	63	(7)	63	(5)	0.957
BMI (kg/m^2^)	26	25 – 30	27	24 – 30	26	25 – 29	0.725
	n	%	n	%	n	%	
Smoking	9	12.3	7	12.7	2	11.1	1.000
Comorbidities							
Hypertension	32	43.8	20	36.4	12	66.7	**0.025**
CAD	3	4.1	2	3.6	1	5.6	1.000
Diabetes	5	6.8	4	7.3	1	5.6	1.000
Medication							
ACE/ARBs	22	30.1	13	23.6	9	50.0	**0.034**
Beta-blockers	13	17.8	8	14.5	5	27.8	0.286
ASA	7	9.6	4	7.3	3	16.7	0.353
Statins	13	17.8	10	18.2	3	16.7	1.000
Aromatase inhibitors	27	37.0	20	36.4	7	38.9	0.847
Tamoxifen	5	6.8	2	3.6	3	16.7	0.092
Radiation doses	Median	Q_1_-Q_3_	Median	Q_1_-Q_3_	Median	Q_1_-Q_3_	
Dmean heart (Gy)	2.2	0.9 – 3.9	3.3	1.8 – 4.2	0.6	0.5 – 0.7	**<0.001**
Dmax heart (Gy)	45.9	7.9 – 47.9	46.9	44.9 – 48.6	4.5	3.7 – 6.3	**<0.001**
V20 Gy to heart (%)	2.0	0.0 – 5.1	4.1	1.4 – 6.1	0	0	**<0.001**
V45 Gy to heart (%)	0.1	0.0 – 0.9	0.6	0.0 – 1.1	0	0	**<0.001**
Dmean LV (Gy)	3.8	1.1 – 5.7	4.4	2.7 – 7.0	0.1	0.1 – 0.2	**<0.001**
Dmax LV (Gy)	44.3	4.6 – 47.0	45.8	42.1 – 47.8	0.4	0.3 – 0.6	**<0.001**
V20 Gy LV (%)	3.2	0.0 – 8.6	6.7	1.8 – 11.4	0	0	**<0.001**
V45 Gy LV (%)	0	0.0 – 0.9	0.2	0.0 – 1.3	0	0	**<0.001**
Dmean RV (Gy)	1.9	1.0 – 3.0	2.4	1.5 – 3.3	0.5	0.3 – 0.8	**<0.001**
Dmean LAD (Gy)	11.5	1.7 – 26.7	21.4	9.4 – 28.6	0.1	0.1 – 0.3	**<0.001**
Dmax LAD (Gy)	42.5	4.5 – 46.4	45.3	37.2 – 46.9	0.4	0.4 – 0.5	**<0.001**
V20 Gy LAD (%)	20.3	0.0 – 61.6	42.7	9.0 – 67.9	0	0	**<0.001**
V45 Gy LAD (%)	0	0.0 – 7.5	0.3	0.0 – 14.3	0	0	**<0.001**

Hypertension (p = 0.025) and the use of angiotensin-converting enzyme inhibitors or angiotensin receptor blockers (p = 0.034) were more common among patients with right-sided disease. As expected, radiation doses to cardiac structures were significantly higher in left-sided cases (p < 0.001).

### Cardiac biomarkers

**Fig. 1:**
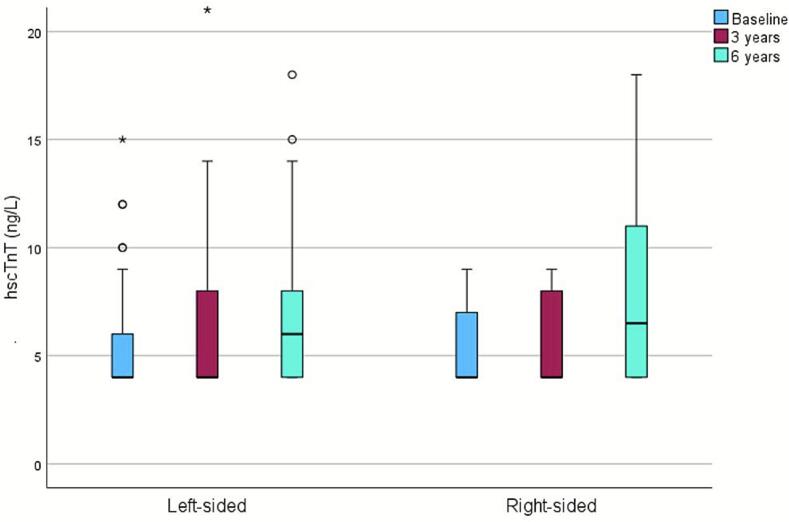
High-sensitivity cardiac troponin T (hscTnT) levels during the follow-up. Outliers and extreme values are presented with circles and asterisks. As the median at baseline and 3 years corresponds to the lowest value used in the analyses (4 ng/L), it overlaps with the lower border of the box.

Levels of hscTnT increased significantly from a median of 4 ng/L (4–7) at baseline to 6 ng/L (4–9) at six years (p < 0.001). This increase was observed only in patients with left-sided disease (p < 0.001), with no significant change in right-sided cases (p = 0.072; Fig. 1). Among left-sided patients, the increase in hscTnT correlated with higher radiation doses to cardiac structures, including Dmax to the left anterior descending artery (LAD) (ρ = 0.288, p = 0.035), Dmax heart (ρ = 0.303, p = 0.026), and V45 heart (ρ = 0.281, p = 0.039).

**Fig. 2:**
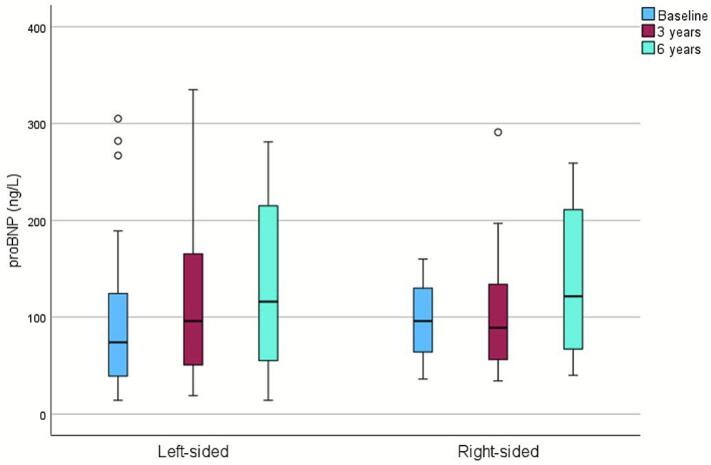
N-terminal pro-brain natriuretic peptide (proBNP) levels during the follow-up. Outliers are presented with circles.

In addition, proBNP increased significantly over time, rising from 78 ng/L (43–127) to 118 ng/L (59–214) at six years (p < 0.001). In left-sided patients, this increase was already significant at three years and persisted for the whole of the follow-up (p < 0.001), while in right-sided patients, it became significant only at six years (p = 0.035; Fig. 2). No significant correlation was found between proBNP and radiation dose parameters.

### Echocardiographic measurements

Table 2, Table 3, and supplementary Table S1 show the echocardiographic measurements for the whole study population, left- and right-sided patients, respectively, at baseline and at three and six years. Baseline echocardiographic parameters were generally well balanced between patients with left- and right-sided breast cancer, except for a slightly longer deceleration time in the left-sided group (p = 0.035). No significant changes were observed in left ventricular (LV) diameters over time.

**Table 2:** Echocardiography measurements of all 73 patients at baseline, at three years and six years.

**LV measurements**	**Baseline mean (SD)**		**Three years mean (SD)**		**Six years mean (SD)**		***p* Value baseline to three years**	***p* Value baseline to six years**	***p* Value three years to six years**
LVEDD (mm)	45	(4)	45	(5)	45	(4)	0.921	0.678	0.689
LVESD (mm)	30	(4)	30	(3)	31	(4)	0.810	0.558	0.604
IVS (mm)	10	(1)	10	(1)	10	(1)	0.242	0.708	0.493
PW (mm)	10	(1)	10	(1)	10	(1)	0.816	0.263	0.254
**LV Systolic function**									
GLS (%)	−18	(3)	−17	(3)	−17	(2)	**0.015**	0.066	0.422
LVEF (%)	64	(8)	59	(7)	60	(7)	**<0.001**	**0.001**	0.373
SV (mL)	73	(16)	68	(13)	67	(12)	**0.007**	**0.014**	0.606
**LV diastolic function**									
IVRT (ms)	107	(26)	114	(22)	115	(26)	**0.025**	**0.044**	0.652
Dt (ms)	227	(42)	236	(57)	234	(65)	0.242	0.445	0.840
Mitral E (cm/s)	73.3	(16.2)	69.6	(16.4)	72.1	(17.1)	**0.011**	0.324	0.125
EA	1.0	(0.3)	1.0	(0.3)	1.0	(0.3)	0.777	0.640	0.754
LAVI (mL/m2)	31.8	(8.7)	34.1	(8.9)	35.1	(14.1)	**0.007**	**0.006**	0.354
LA EF (%)	58	(8)	55	(11)	53	(14)	**0.017**	**0.007**	0.247
Conduit fraction (%)	28	(8)	24	(10)	24	(10)	**0.005**	0.060	0.861
Active pump fraction (%)	42	(10)	42	(9)	41	(13)	0.948	0.257	0.299
**RV function**									
TAPSE (mm)	24	(4)	23	(4)	23	(5)	0.071	0.383	0.646
RV*s*′ (cm/s)	12.5	(3.0)	12.4	(2.6)	12.6	(3.0)	0.828	0.792	0.590
RV*Ee*′	4.2	(1.3)	4.2	(1.5)	4.0	(1.4)	0.607	0.312	0.065
TI gradient (mmHg)	22	(6)	24	(7)	25	(7)	**0.003**	**<0.001**	0.243
SD, standard deviation; LV, left ventricular; LVEDD, left ventricular end-diastolic dimension; LVESD, left ventricular end-systolic dimension; IVS, interventricular septum thickness; PW, posterior wall thickness; GLS, global longitudinal strain; EF, ejection fraction; IVRT: isovolumetric relaxation time; Dt, deceleration time; Mitral E, first peak of diastole, active filling; EA, ratio of diastolic peaks E and A; LAVI, left atrial volume at the end-systole; LA, left atrium; RV, right ventricle; TAPSE, tricuspid annular plane systolic excursion; RV s′, right ventricular systolic velocity of pulsed tissue Doppler; RV Ee′, ratio of early transtricuspidal flow velocity (E) to early diastolic velocity of the tricuspid valve annulus (e′); TI, maximum tricuspid regurgitation gradient.

**Table 3:** Echocardiography measurements of left-sided patients (n = 55) at baseline, and at three years and six years.

**LV measurements**	**Baseline mean (SD)**		**Three years mean (SD)**		**Six years mean (SD)**		***p* Value baseline to three years**	***p* Value baseline to six years**	***p* Value three years to six years**
LVEDD (mm)	45	(4)	45	(4)	45	(4)	1.000	0.423	0.507
LVESD (mm)	30	(4)	30	(3)	31	(3)	0.863	0.248	0.139
IVS (mm)	10	(1)	10	(2)	10	(1)	0.424	0.914	0.455
PW (mm)	10	(1)	10	(2)	10	(1)	0.786	0.495	0.430
**LV Systolic function**									
GLS (%)	−18	(3)	−17	(3)	−17	(2)	**0.001**	**0.006**	0.275
LVEF (%)	65	(7)	60	(7)	60	(7)	**<0.001**	**0.002**	0.591
SV (mL)	74	(15)	69	(14)	67	(11)	**0.011**	**0.015**	0.738
**LV diastolic function**									
IVRT (ms)	104	(25)	113	(24)	115	(28)	**0.007**	**0.024**	0.704
Dt (ms)	233	(41)	240	(58)	238	(68)	0.419	0.687	0.860
Mitral E (cm/s)	72.6	(14.7)	69.2	(15.6)	71.6	(16.3)	**0.063**	0.554	0.192
EA	1.0	(0.3)	1.0	(0.3)	1.0	(0.3)	0.753	0.899	0.942
LAVI (mL/m2)	32.1	(8.3)	35.0	(8.8)	35.9	(13.7)	**0.007**	**0.009**	0.391
LA EF (%)	58	(9)	54	(11)	52	(15)	**0.018**	**0.006**	0.408
Conduit fraction (%)	27	(8)	22	(9)	23	(10)	**0.007**	**0.036**	0.572
Active pump fraction (%)	42	(10)	42	(9)	40	(13)	0.780	0.256	0.265
**RV function**									
TAPSE (mm)	24	(4)	23	(4)	24	(5)	0.201	0.787	0.271
RV *s*′ (cm/s)	12.3	(3.1)	12.4	(2.7)	12.7	(2.9)	0.812	0.466	0.469
RV *Ee*′	4.2	(1.2)	4.1	(1.4)	3.8	(1.1)	0.728	0.076	0.129
TI gradient (mmHg)	22	(6)	24	(6)	25	(7)	**0.013**	**0.009**	0.355
For abbreviations, see Table 2.									

### Left ventricular systolic function

LV ejection fraction (LVEF), global longitudinal strain (GLS), and stroke volume (SV) were used to assess LV systolic function. All three parameters worsened significantly from baseline to three years in the overall population: LVEF, GLS, and SV deteriorated by 5 (10)% (p < 0.001), 1 (4)% (p = 0.015), and 5 (15)ml (p = 0.007), respectively. The impairment persisted at six years in LVEF (p = 0.001) and SV (p = 0.014). These changes were restricted to patients with left-sided disease. In this subgroup LVEF declined from 65 (7)% to 60 (7)% (p = 0.002), GLS from −18 (3)% to −17 (2)% (p = 0.006), and SV from 74 (15)ml to 67 (11)ml (p = 0.015). No significant changes in LV systolic function were observed in right-sided patients.

Multivariable analysis showed that AI use (β = 0.415, p = 0.001) and left-sided disease (β = 0.380, p = 0.013) predicted a greater GLS impairment at three years. Higher BMI predicted a decrease in SV (β = -0.323, p = 0.031). No correlation was found between cardiac radiation dose and LV systolic function impairment.

### Left ventricular diastolic function

Worsening in LV diastolic function during the entire follow-up was seen across the full cohort. Isovolumic relaxation time (IVRT) prolonged by 8 (32) ms (p = 0.044), the maximal left atrial (LA) volume indexed to the patient’s body surface area (LAVI) increased by 3.5 (10.2) mL/m^2^ (p = 0.006), and the LA ejection fraction (LA EF) decreased by 5 (14) % (p = 0.007). At three years, the first peak of diastole (mitral E) was also significantly decreased by 4.1 (13.5) cm/s (p = 0.011) and the passive conduit fraction of LA EF dropped by 4 (10) % (p = 0.005). These changes were driven by left-sided patients; right-sided patients showed no significant changes in diastolic parameters.

In a multivariable analysis, a higher MHD predicted an increase (β = 0.395, p = 0.014) and BMI decrease (β = -0.388, p = 0.009) in IVRT from baseline to six years. Furthermore, hypertension (β = 0.284, p = 0.039) and smoking (β = -0.288, p = 0.017) predicted mitral E changes. In addition, AI use (β = -0.493, p < 0.001) and MHD (β = -0.408, p = 0.010) were predictive of a decrease in LA EF. AI use also predicted a decline in active pump fraction (β = -0.333, p = 0.025).

### Right ventricular function

Right ventricular (RV) changes were less pronounced. The tricuspid regurgitation pressure gradient (TI gradient) increased by 3 (7) mmHg (p < 0.001) across the cohort. In right-sided patients, the ratio of early transtricuspidal flow velocity to early diastolic velocity of the mitral valve annulus (RV Ee′) increased significantly from 4.1 (1.7) to 4.6 (2.0) (p = 0.043), while in left-sided patients, a non-significant decline was observed (p = 0.076). Tricuspid annular plane systolic excursion (TAPSE) remained unchanged.

In a multivariable analysis, AI use was predictive of a decrease in TAPSE (β = -0.313, p = 0.023), whereas having a left-sided tumor was predictive of a decline in RV Ee′ (β = -0.332, p = 0.036).

## Discussion

**Fig. 3:**
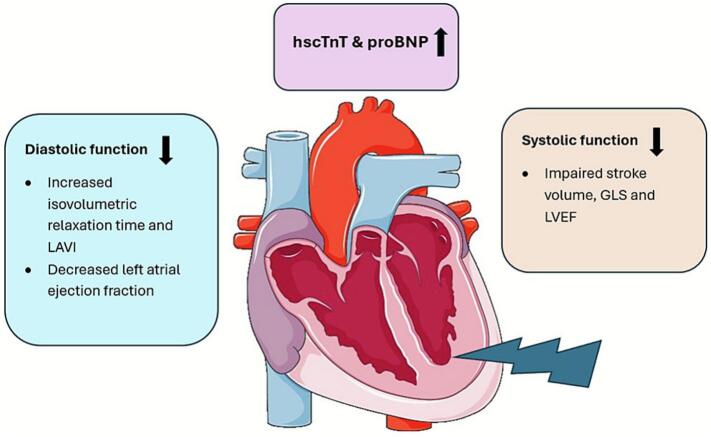
Summary of the study results. Subclinical deterioration of systolic and diastolic function was observed during the six-year follow-up after breast radiotherapy. Furthermore, the levels of hscTnT and proBNP increased.

This six-year prospective study demonstrates that subclinical cardiac changes—both functional and biochemical—can emerge and persist following adjuvant RT for early breast cancer (Fig. 3). Specifically, we observed significant increases in cardiac biomarkers (hscTnT, proBNP), as well as declines in LV systolic and diastolic function, primarily in patients with left-sided disease. These findings suggest that radiation-induced cardiotoxicity can develop early and remain stable over time, indicating potentially permanent cardiac alterations.

### Cardiac biomarkers

We observed a significant hscTnT increase, attributed to the left-sided patients, which was most robust between three and six years. Furthermore, a correlation with higher RT doses was seen in patients with left-sided cancer.

It is shown that myocardial injury elevates serum hscTnT levels, and thus, serum hscTnT is used to detect acute or chronic myocardial damage [13]. In our previous study, we showed that patients with a significant increase in hscTnT during RT had higher cardiac radiation doses attributed to the acute cardiotoxicity of RT [14]. Furthermore, the study by Darby et al. showed that long-term rates of major coronary events increased linearly with the MHD by 7.4 % per Gy [7]. While myocardial damage increases with higher radiation doses and the cardiac dose is unevenly distributed, the dose received in cardiac substructures may be more reliable in predicting long-term cardiotoxicity [15]. This aligns with our finding of a correlation between hscTnT increase during the long-term follow-up and greater radiation doses received. Since MHD does not adequately predict doses received by cardiac substructures, especially LV and LAD, accumulating high doses, they should be individually assessed [15,16]. Future studies are called for to determine the safety limits of radiation doses for significant cardiac substructures.

Natriuretic peptides, especially proBNP, are used to evaluate acute and chronic heart failure [13]. The synthesis of proBNP is induced by volume and pressure overload in the ventricles as a marker for mechanical stress [13]. Previously, Saiki et al. [17] reported an 8 % incidence of heart failure within six years after breast cancer RT, which is in line with our findings of a significant increase in proBNP after a six-year follow-up. Furthermore, additional studies have reported that proBNP increases after RT, and there is an association, especially with high radiation doses received [[18], [19], [20]]. These findings endorse our results for the utility of proBNP assessment after breast RT.

### Left ventricle systolic function

This study evaluated LV systolic function using LVEF, GLS, and SV. Importantly, systolic impairment was predominantly seen in patients with left-sided disease, consistent with their higher cardiac radiation exposure. However, we did not observe a direct association between radiation dose parameters and the magnitude of LVEF or GLS decline in multivariable analyses. This may reflect either a threshold effect or individual patient susceptibility, which should be explored in larger cohorts.

In our previous work, we also demonstrated early impairment in GLS immediately after RT, with delayed deterioration seen in LVEF and SV [10], a trajectory that is reinforced by this extended follow-up. GLS is the most sensitive and reproducible measure and is considered the optimal deformation parameter for detecting subclinical LV dysfunction preceding LVEF decrease, allowing early intervention [21]. Multiple trials have shown the utility of GLS in cancer patients; however, most studies are conducted with patients also receiving chemotherapy [21].

The CAROLE study investigated a multimodality approach, including GLS measurement for cardiotoxicity screening in long-term breast cancer survivors [22]. A total of 203 breast cancer patients, most treated over ten years earlier, were studied. Of all, 31.5 % had received only adjuvant RT, and 8.5 % had received RT and adjuvant chemotherapy. Overall, 77.6 % of the patients had preclinical or clinical cardiovascular disease (CVD), and interestingly, the GLS measurement identified 13 % of CVD that otherwise would have been missed. Furthermore, Trivedi et al. [23] reported a decline in GLS in breast cancer patients up to 12 months after RT. Similar to our findings, no change in conventional echocardiography was observed during the early follow-up. These findings reinforce GLS imaging as a beneficial method for better detecting early changes in heart function after RT.

Obesity, as reflected by higher BMI, was linked to a greater reduction in SV, consistent with its known role as a risk factor for heart failure [24]. These observations support the need for a multimodal risk assessment approach in breast cancer patients receiving adjuvant therapy, integrating treatment-related factors and baseline cardiovascular risk.

### Diastolic changes

Heart failure with preserved ejection fraction (HFpEF) is a multifactorial syndrome caused by systemic microvascular inflammation and coronary microvascular dysfunction, leading to fibrosis, myocardial stiffening, and diastolic dysfunction characterized by clinical symptoms, distinctively dyspnea, and fatigue, while the EF remains normal [25]. RT induces inflammation, oxidative stress, and chronic gene expression changes, causing fibrosis, which increases the risk of HFpEF [26,27]. However, prospective studies addressing the development of HFpEF after RT are lacking. We found a significant worsening of LV diastolic function during the six-year follow-up after RT. Similarly, for systolic function, the decrease was accounted for in left-sided patients.

While most of the previous research has focused on heart failure with reduced ejection fraction (HFrEF), it seems that HFpEF is indeed more prevalent among breast cancer survivors [28]. In fact, in a population-based case-control study of incident HF conducted by Saiki et al. [17], they observed an up to 16-fold relative risk increase of HFpEF but not HFrEF with increasing cardiac radiation exposure during breast cancer RT after a long-term follow-up. In addition, in a case-control study of almost 90,000 women by Kwan et al. [29], the 10-year cumulative incidence rate for HFpEF was 0.8 % compared to 0.4 % for HFrEF after breast cancer treatment. These findings align with our results and highlight the subclinical nature of diastolic dysfunction in this population and the value of early detection through advanced echocardiographic measures.

### Aromatase inhibitor effects

AIs are a part of the standard treatment for most post-menopausal breast cancer patients, recommended for all luminal-like breast cancers as an adjuvant treatment [4]. However, using AIs also seems to reduce endothelial function, which is a predictor of CVD [30]. Furthermore, AI use has been linked to an increased risk for CVD, including ischemic heart disease, heart failure, and arrhythmias, especially compared to tamoxifen therapy [[31], [32], [33], [34], [35]]. On the other hand, some evidence suggests that the difference may be driven by the cardioprotective effects of tamoxifen [32]. The duration of the AI therapy appears essential, as more extended use seems more harmful [34].

In our study, AI use was adversely associated with changes in systolic (GLS, TAPSE) and diastolic (LA EF, active pump fraction) function parameters. Interestingly, AI use was independently associated with GLS impairment at three years, but this association was no longer observed at six years. This temporal pattern may reflect partial recovery of myocardial strain following cessation of AI therapy, which typically ends at five years. A similar delayed recovery pattern has been noted in our earlier bone mineral density studies in similar patient population [36].

## Limitations

Although our study benefits from a prospective design and a relatively long follow-up, it is not without limitations. The lack of a non-irradiated control group limits our ability to isolate radiation effects from natural aging or other comorbidities. Additionally, the sample size—particularly of right-sided cases—was modest, which may affect statistical power for subgroup analyses.

## Conclusion

Adjuvant breast cancer radiotherapy led to persistent subclinical impairment in cardiac function, along with elevated biomarkers over a six-year period. Higher radiation doses and concurrent AI therapy were associated with more pronounced changes. Our findings emphasize the value of integrating longitudinal cardiac monitoring — especially GLS and biomarkers — into follow-up protocols for patients receiving RT. Further research is needed to define thresholds for intervention and to explore cardioprotective strategies to mitigate these effects.

## Funding

This study received funding from non-profit trusts: Paavo and Eila Salonen Legacy, Georg and Ella Ehrnrooth Legacy, Aarne Koskelo Legacy, Elli and Elvi Oksanen Legacy, Finnish Cultural Foundation Pirkanmaa Regional fund and the Competitive State Research Financing of the Expert Responsibility area of Tampere University Hospital. The funding sources were not involved in the study design, analysis, or reporting.
