# Supplementary material for: The evolution of cardiac changes after breast cancer adjuvant radiotherapy – A six-year follow-up study
Source: Clin Transl Radiat Oncol. 2025 Nov 15;56:101078. doi: 10.1016/j.ctro.2025.101078 (PMC12666111; doi:10.1016/j.ctro.2025.101078)
Supplement: Supplementary Data 1 [file mmc1.pdf]

Table S1. Echocardiography measurements of right-sided patients (n=18) at baseline, and at three years and six years

	Baseline mean (SD)		Three years mean (SD)		Six years mean (SD)		*p* Value* baseline to three years	*p* Value* baseline to six years	*p* Value* three years to six years
**LV measurements**									
LVEDD (mm)	44	(5)	44	(5)	44	(5)	0.763	0.474	0.567
LVESD (mm)	30	(3)	31	(3)	30	(5)	0.305	0.519	0.195
IVS (mm)	10	(2)	10	(1)	10	(1)	0.368	0.368	1.000
PW (mm)	10	(1)	10	(2)	10	(1)	1.000	0.286	0.381
**LV Systolic function**									
GLS	-17	(4)	-18	(3)	-17	(3)	0.570	0.557	0.901
LVEF (%)	64	(10)	60	(7)	61	(6)	0.055	0.151	0.345
SV (mL)	69	(17)	67	(12)	64	(15)	0.421	0.425	0.680
**LV diastolic function**									
IVRT (ms)	116	(27)	114	(14)	115	(21)	0.706	0.902	0.805
Dt (ms)	209	(43)	223	(54)	221	(54)	0.359	0.347	0.926
Mitral E (cm/s)	77.1	(20.1)	70.9	(19.2)	73.3	(19.8)	0.073	0.375	0.438
EA	1.0	(0.3)	1.0	(0.3)	0.9	(0.2)	0.165	0.172	0.479
LAVI (mL/m2)	30.8	(10.0)	31.7	(8.7)	32.7	(15.4)	0.538	0.387	0.720
LA EF (%)	59	(6)	60	(8)	57	(12)	0.707	0.776	0.362
Conduit fraction (%)	30	(9)	29	(10)	27	(9)	0.460	0.955	0.483
Active pump fraction (%)	41	(9)	44	(9)	43	(12)	0.612	0.860	0.981
**RV function**									
TAPSE (mm)	24	(5)	23	(4)	22	(6)	0.197	0.117	0.412
RV *s*' (cm/s)	13.0	(2.7)	12.5	(2.4)	12.4	(3.5)	0.341	0.479	0.927
RV *Ee*'	4.1	(1.7)	4.7	(1.8)	4.6	(2.0)	**0.021**	**0.043**	0.298
TI gradient (mmHg)	23	(7)	26	(7)	27	(6)	0.125	**0.003**	0.429

SD, standard deviation; LV, left ventricular; LVEDD, left ventricular end-diastolic dimension; LVESD, left ventricular end-systolic dimension; IVS, interventricular septum thickness; PW, posterior wall thickness; GLS, global longitudinal strain; EF, ejection fraction; IVRT: isovolumetric relaxation time; Dt, deceleration time; Mitral E, first peak of diastole, active filling; EA, ratio of diastolic peaks E and A; LAVI, left atrial volume at the end-systole; LA, left atrium; RV, right ventricle; TAPSE, tricuspid annular plane systolic excursion; RV s', right ventricular systolic velocity of pulsed tissue Doppler; RV Ee', ratio of early transtricuspidal flow velocity (E) to early diastolic velocity of the tricuspid valve annulus (e'); TI, maximum tricuspid regurgitation gradient.
